# Quantitative Analysis of the Anti-Proliferative Activity of Combinations of Selected Iron-Chelating Agents and Clinically Used Anti-Neoplastic Drugs

**DOI:** 10.1371/journal.pone.0088754

**Published:** 2014-02-20

**Authors:** Eliska Potuckova, Hana Jansova, Miloslav Machacek, Anna Vavrova, Pavlina Haskova, Lucie Tichotova, Vera Richardson, Danuta S. Kalinowski, Des R. Richardson, Tomas Simunek

**Affiliations:** 1 Department of Biochemical Sciences, Faculty of Pharmacy in Hradec Králové, Charles University in Prague, Prague, Czech Republic; 2 Molecular Pharmacology and Pathology Program, Bosch Institute and Department of Pathology, University of Sydney, Sydney, Australia; Indiana University School of Medicine, United States of America

## Abstract

Recent studies have demonstrated that several chelators possess marked potential as potent anti-neoplastic drugs and as agents that can ameliorate some of the adverse effects associated with standard chemotherapy. Anti-cancer treatment employs combinations of several drugs that have different mechanisms of action. However, data regarding the potential interactions between iron chelators and established chemotherapeutics are lacking. Using estrogen receptor-positive MCF-7 breast cancer cells, we explored the combined anti-proliferative potential of four iron chelators, namely: desferrioxamine (DFO), salicylaldehyde isonicotinoyl hydrazone (SIH), (*E*)-*N′*-[1-(2-hydroxy-5-nitrophenyl)ethyliden] isonicotinoyl hydrazone (NHAPI), and di-2-pyridylketone 4,4-dimethyl-3-thiosemicarbazone (Dp44mT), plus six selected anti-neoplastic drugs. These six agents are used for breast cancer treatment and include: paclitaxel, 5-fluorouracil, doxorubicin, methotrexate, tamoxifen and 4-hydroperoxycyclophosphamide (an active metabolite of cyclophosphamide). Our quantitative chelator-drug analyses were designed according to the Chou-Talalay method for drug combination assessment. All combinations of these agents yielded concentration-dependent, anti-proliferative effects. The hydrophilic siderophore, DFO, imposed antagonism when used in combination with all six anti-tumor agents and this antagonistic effect increased with increasing dose. Conversely, synergistic interactions were observed with combinations of the lipophilic chelators, NHAPI or Dp44mT, with doxorubicin and also the combinations of SIH, NHAPI or Dp44mT with tamoxifen. The combination of Dp44mT with anti-neoplastic agents was further enhanced following formation of its redox-active iron and especially copper complexes. The most potent combinations of Dp44mT and NHAPI with tamoxifen were confirmed as synergistic using another estrogen receptor-expressing breast cancer cell line, T47D, but not estrogen receptor-negative MDA-MB-231 cells. Furthermore, the synergy of NHAPI and tamoxifen was confirmed using MCF-7 cells by electrical impedance data, a mitochondrial inner membrane potential assay and cell cycle analyses. This is the first systematic investigation to quantitatively assess interactions between Fe chelators and standard chemotherapies using breast cancer cells. These studies are vital for their future clinical development.

## Introduction

Breast cancer is a common malignancy and is one of the leading causes of cancer deaths among women [1]. An accumulating body of evidence suggests a role for iron (Fe) in the etiology of breast cancer (reviewed in [2]). Indeed, rapidly growing and dividing cancer cells have a higher requirement for Fe (reviewed in [3], [4]) and breast cancer cells possess multiple mechanisms to increase cellular Fe levels. For example, breast cancer cells: **(1)** express high levels of transferrin receptor 1 on their surface [5]; **(2)** demonstrate decreased expression of the Fe efflux protein, ferroportin1 [6]; and **(3)** synthesize transferrin, which is stimulated by estrogen receptor signaling and may increase Fe uptake *via* an autocrine mechanism [7]. Interestingly, several studies have shown that Fe levels are increased in breast cancer tumors compared to normal breast tissue [8], [9] and a positive correlation has been reported between Fe content and the aggressiveness of the tumor [10]. Furthermore, high Fe levels have been identified as a risk factor for breast cancer development [11], [12].

For many years, Fe chelators such as desferrioxamine (DFO) have been successfully used in clinical practice for the management of Fe overload disease, including β-thalassemia major [13]. In such conditions, chelators promote Fe excretion and act to protect against the toxicity of Fe that is induced *via* oxidative injury (reviewed in [14]). More recently, a new possible use of novel Fe chelators has emerged, as they have shown potential in anti-cancer treatment [14]–[17]. Previously, several Fe chelators have been demonstrated to selectively trigger apoptotic cell death in MCF-7 breast cancer cells, while sparing non-cancerous cells, such as normal human mammary epithelial cells, fibroblasts, or cardiomyoblasts [18]–[20]. In addition, Fe chelation has been suggested to prevent or alleviate the side effects of several chemotherapeutic agents, such as the cardiotoxicity associated with anthracyclines [21], [22].

**Figure 1:**
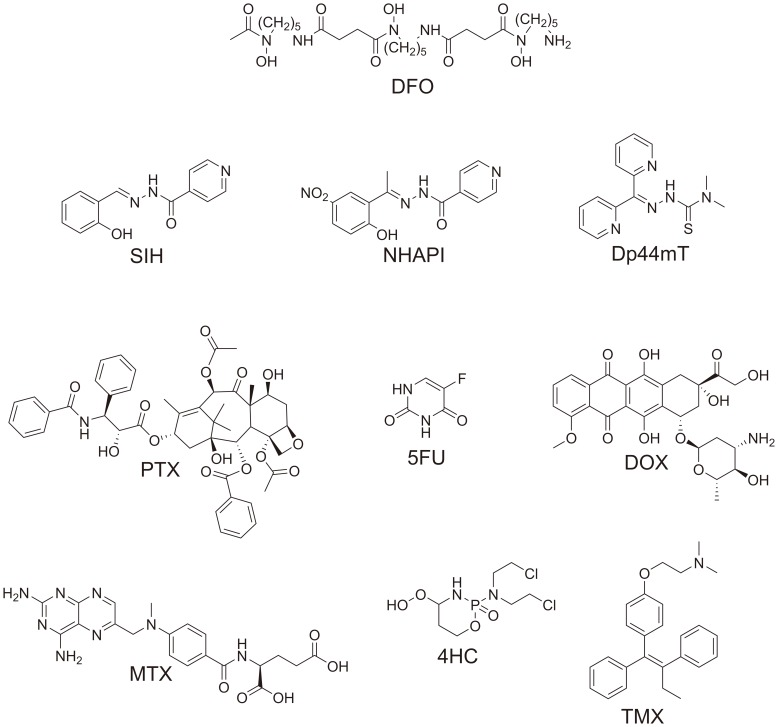
Line drawings of the structures of the studied compounds. Metal chelators: desferrioxamine (DFO); salicylaldehyde isonicotinoyl hydrazone (SIH); (*E*)-*N′*-[1-(2-hydroxy-5-nitrophenyl)ethyliden] isonicotinoyl hydrazone (NHAPI); and di-2-pyridylketone 4,4-dimethyl-3-thiosemicarbazone (Dp44mT). Anti-neoplastic agents: paclitaxel (PTX); 5-fluorouracil (5FU); doxorubicin (DOX); methotrexate (MTX); 4-hydroperoxycyclophosphamide (4HC); and tamoxifen (TMX).

However, before Fe chelators can be introduced into chemotherapy protocols, it is essential to establish how these compounds potentially promote or interfere with the anti-tumor effects of other chemotherapeutic agents. Unfortunately, these data are lacking for the majority of drugs currently used for breast cancer treatment. Hence, in the present study, we investigated the combinatory effects of four diverse Fe chelators and six well-established anti-neoplastic drugs (Fig. 1) using the MCF-7 breast adenocarcinoma cell line. This analysis was achieved using the Chou-Talalay method for the quantitative analysis of drug combinations [23]. Both the chelators and anti-cancer agents were chosen to cover a broad spectrum of characteristics and mechanisms of action.

Notably, DFO is a bacterial siderophore with limited membrane permeability that has been used for decades to manage Fe overload (reviewed in [14]) and has also been extensively tested as a potential anti-cancer agent [24]–[26]. Salicylaldehyde isonicotinoyl hydrazone (SIH; Fig. 1) is a membrane-permeable aroylhydrazone Fe chelator that has demonstrated considerable potential to protect various cell-types against oxidative injury [27]–[29]. Interestingly, SIH has been shown to differentially modulate anthracycline toxicity in cardiac and cancer cells [30]. At concentrations where SIH significantly reduced daunorubicin toxicity in rat cardiomyocytes, this chelator augmented the anti-proliferative activity of daunorubicin against the promyelocytic leukemia cell line, HL-60 [30]. In addition to these previously studied ligands, we also used a novel SIH derivative, (*E*)-*N′*-[1-(2-hydroxy-5-nitrophenyl)ethyliden] isonicotinoyl hydrazone (NHAPI; Fig. 1), which has increased stability against plasma hydrolysis [31], improved anti-proliferative activity against breast cancer cells and reduced toxicity towards cardiomyocytes [18]. Moreover, we also tested the membrane permeable, redox-active ligand, di-2-pyridylketone 4,4-dimethyl-3-thiosemicarbazone (Dp44mT; Fig. 1), which shows marked anti-proliferative activity *in vitro* and *in vivo* [32]–[35].

The four chelators, DFO, SIH, NHAPI and Dp44mT, were assayed in combination with six conventional anti-cancer agents that are commonly used in breast cancer chemotherapy regimens [36], [37]. These six drugs included: the mitotic inhibitor, paclitaxel (PTX), the pyrimidine analog/anti-metabolite, 5-fluorouracil (5FU), the topoisomerase IIα poison, doxorubicin (DOX), the inhibitor of folic acid metabolism, methotrexate (MTX), the estrogen receptor antagonist, tamoxifen (TMX), and 4-hydroperoxycyclophosphamide (4HC), which is an active metabolite of cyclophosphamide, a nitrogen mustard alkylating agent [38].

## Materials and Methods

### 1. Chemicals

DFO was purchased from Novartis (Basel, Switzerland). SIH, NHAPI and Dp44mT were synthesized and characterized, as described previously [31], [35], [39] and their identities and purities were confirmed using elementary analysis (*i.e.*, C, N, H, S), ^1^H and ^13^C NMR and IR spectroscopy. PTX was purchased from Cedarburg Hauser Pharmaceuticals (Denver, U.S.A.), 5FU was from Sandoz (Holzkirchen, Germany), MTX was obtained from Hospira (Lake Forrest, U.S.A.), DOX was from Teva Pharmaceuticals (Petach Tikva, Israel), TMX was from Sigma-Aldrich (Schnelldorf, Germany) and 4HC was from Santa Cruz Biotechnology (Santa Cruz, U.S.A.). All the pharmaceutical products comply with European Pharmacopoeia. For exact dosing, only injectable forms were used. Constituents of various buffers, as well as other chemicals, were obtained from Sigma-Aldrich (Schnelldorf, Germany), Fluka (Seelze, Germany) or Penta (Prague, Czech Republic), and were of the highest pharmaceutical or analytical grade available.

### 2. Cell Culture

The MCF-7 and MDA-MB-231 human breast adenocarcinoma cell lines and T47D human ductal breast carcinoma cell line were purchased from the European Collection of Cell Cultures (ECACC). The cells were cultured in Dulbecco’s modified Eagle’s medium with (MDA-MB-231) or without (MCF-7 and T47D) phenol red (DMEM, Lonza, Belgium), supplemented with 10% heat-inactivated fetal bovine serum (FBS; Lonza), 4 mM L-glutamine (Lonza), 1% penicillin/streptomycin solution (Lonza) and 10 mM HEPES buffer (Sigma, Germany). The medium without phenol red was used for the two cells lines expressing the estrogen receptor (*i.e.*, MCF-7 and T47D) due to the potential influence of phenol red on estrogen signaling [40]. The cells were cultured in 75 cm^2^ tissue culture flasks (TPP, Switzerland) at 37°C in a humidified atmosphere containing 5% CO_2_. Sub-confluent cells were sub-cultured every 3–4 days. For proliferation experiments, the cells were seeded in 96-well plates (TPP) at a density of 5,000 cells/well 24 h prior to the addition of each agent or their combinations. For flow cytometric analyses, the MCF-7 cells were seeded in 60-mm Petri dishes (TPP) at a density of 210,000 cells/well. For microscopic assessments, the MCF-7 cells were seeded in 12-well plates (TPP) at a density of 35,000 cells/well.

### 3. Proliferation Studies

The anti-proliferative effects of the studied agents and their combinations were assayed after a 72 h incubation at 37°C. The lipophilic compounds (SIH, NHAPI, Dp44mT, PTX and TMX) were dissolved in dimethyl sulfoxide (DMSO; Sigma) and then the solution diluted using culture medium so that the final concentration was 0.1% (*v/v*). At this concentration, DMSO had no effect on cellular proliferation (data not shown). To assess the metal complexes, metal salts (ammonium Fe citrate (Sigma) or Cu sulfate (Penta)) were added to cell culture medium before the addition of the chelator in either a 2∶1 (SIH, NHAPI, and Dp44mT) or 1∶1 (DFO) chelator-metal ratio, *i.e.*, at a metal-binding equivalent ratio of 1 [14].

Cellular viability was determined using the neutral red (NR) uptake assay, which is based on the ability of viable cells to incorporate NR (Sigma) into lysosomes [31], [41]. The optical density of soluble NR was measured at λ  = 540 nm using a Tecan Infinite 200 M plate reader (Tecan, Austria). Manual cell counts demonstrated that absorption was directly correlated to cell number. The proliferation of cells in the experimental groups was expressed as a percentage of the untreated control (100%).

Combination studies were designed according to the method described by Chou and Talalay [23]. Briefly, the concentration of chelator or anti-neoplastic agent that induced a 50% decrease in proliferation (IC_50_) was determined for each agent alone. Then, both the individual agent and the combinations were tested at concentrations corresponding to fractions and multiples (1/8, 1/4, 1/2, 1, 2, and 4) of their IC_50_ values.

The proliferation of MCF-7 cells was also monitored using an xCELLigence System (Roche, Penzberg, Germany), which measures electrical impedance across micro-electrodes that are integrated on the bottom of the tissue culture plates provided by the manufacturer (xCELLigence System). The cell adhesion rate, which is expressed as the “cell index” and parallels the viability or proliferation of the cells, is monitored throughout the entire incubation period [42]. This real-time analysis was performed for selected combinations of NHAPI (or its Fe complex) with TMX.

### 4. Fluorescence Microscopy Assessments

Changes in cellular morphology and the mitochondrial inner membrane potential (ΔΨ_m_) of MCF-7 cells were evaluated using an Eclipse TS100 inverted epifluorescence microscope (Nikon, Japan), that was equipped with a cooled digital camera (1300Q, VDS Vosskühler, Germany), and NIS-Elements AR 2.30 software (Laboratory Imaging, Czech Republic). The cells were seeded in 12-well plates and incubated as described above. The JC-1 probe (5,5′,6,6′–tetrachlor-1,1′,3,3′-tetraetylbenzimidazolylcarbocyanine iodide, Molecular Probes, Eugene, OR, U.S.A.) was used for the ΔΨ_m_ assessments. Notably, JC-1 non-specifically accumulates in the cytosol as a green fluorescent monomer (λ_ex_  = 480 nm; λ_em_  = 535 nm), whereas JC-1 monomers assemble into J-aggregates with red fluorescence (λ_ex_  = 560 nm; λ_em_  = 630 nm) in metabolically active mitochondria with polarized inner membranes. The cells were loaded with JC-1 (2 µM) for 15 min at room temperature and the medium containing JC-1 was then replaced with PBS. Sample images were captured using the microscope set-up outlined above.

### 5. Flow Cytometric Inner Mitochondrial Membrane Potential (ΔΨ_m_) Assessment

MCF-7 cells were seeded in 60-mm Petri dishes and incubated for 72 h at 37°C with the evaluated agents or their combinations. The cells were then harvested by trypsinization and loaded with JC-1 (2 µM) for 15 min at room temperature. The intensities of red (530 nm, FL-1) and green fluorescence (585 nm, FL-2) were measured after JC-1 excitation at 488 nm using an Accuri C6 flow cytometer (Accuri Cytometers Europe Ltd., U.K.). A total of 10,000 events were collected per analysis.

### 6. Cell Cycle Analysis

Following a 72 h/37°C incubation with each agent or the combinations, the MCF-7 cells were harvested by trypsinization, centrifuged at 300 x *g*, washed in PBS supplemented with 5% FBS (PBS+FBS), and suspended in a small amount of PBS+FBS. Ice-cold 70% ethanol was then added in a drop-wise fashion and the cells were fixed for 3 h/−20°C. After fixation, the ethanol was removed by centrifugation and the cells were washed in PBS+FBS and resuspended in sodium citrate (4 mM) in PBS+FBS. The cells were then incubated with 200 µg/mL of RNAse A (Sigma) and 30 µg/mL of propidium iodide (PI) for 20 min/37°C. The cell cycle distributions were measured using an Accuri C6 flow cytometer with PI being excited at 488 nm, and fluorescence detected at 585 nm (FL-2). A total of 10,000 events were collected per analysis.

### 7. Data Analysis and Statistics

SigmaStat for Windows 3.5 (SPSS, U.S.A.) statistical software was used in this study. The data are expressed as the mean ± S.D. of at least 3 experiments. Statistical significance was determined using a one-way ANOVA with a Bonferroni post-hoc test (comparisons of multiple groups against the corresponding control). The results were considered to be statistically significant when *p*<0.05. The IC_50_ values (the concentration of agent inducing a 50% decrease in proliferation compared to the untreated control or the median effective dose) were calculated using CalcuSyn 2.0 software (Biosoft, Cambridge, U.K.) with the following equation:
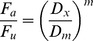
where *F_a_* is the fraction affected (proliferation inhibited) by the drug treatment, *F_u_* is the uninhibited fraction, *D_x_* is the dose of a drug, *D_m_* is the median effect dose (IC_50_) and *m* is the slope of the curve. The software was also used to obtain the combination index (*CI*), a rigorous quantitative measure of the degree of drug interaction, using the following equation:



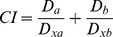
where *D_a_* and *D_b_* are the doses of the drugs that were used in combination, and *D_xa_* and *D_xb_* are the iso-effective doses. Chou and Talalay [23] describe drug interactions in terms of either a nearly additive effect (*CI*: 0.9–1.1), slight synergism (*CI*: 0.85–0.90), moderate synergism (*CI:* 0.7–0.85), synergism (*CI:* 0.3–0.7), strong synergism (*CI:* 0.1–0.3), very strong synergism (CI <0.1), slight antagonism (*CI:* 1.1–1.2), moderate antagonism (*CI:* 1.20–1.45), antagonism (*CI:* 1.45–3.3), strong antagonism (*CI:* 3.3–10), and very strong antagonism (*CI*>10). Furthermore, to assess the changes in drug–drug interactions as a function of concentration or activity, F*a-CI* plots were calculated using CalcuSyn computer simulations (*via* the CalcuSyn 2.0 software described above).

The flow cytometry files were analyzed using Cyflogic software (CyFlo Ltd, Finland) and the cell cycle analyses were performed using MultiCycle AV Software (Phoenix Flow Systems, U.S.A.).

## Results

### 1. The Studied Iron Chelators Show Comparable or Greater Anti-Proliferative Activity than Standard Chemotherapeutic Agents in Clinical Use

In the initial assessment of the anti-proliferative effects of the studied Fe chelators and clinically used anti-neoplastic agents, MCF-7 cells were incubated for 72 h/37°C with an increasing concentration of each of the individual agents. All of the examined chelators and cytotoxic chemotherapeutics displayed a concentration-dependent decrease in cancer cell growth (Figs. S1 and S2). The tested anti-cancer drugs differed greatly in their anti-proliferative efficacy (Table 1), with the IC_50_ values ranging from nanomolar (PTX, IC_50_ = 0.008±0.005 µM) to millimolar concentrations (MTX, IC_50_>3000 µM). Of the chelators used in this study, DFO, SIH, and NHAPI displayed comparable anti-proliferative properties, with IC_50_ values between 14.0–19.1 µM. Significantly, this was >3,000 times higher than the IC_50_ of Dp44mT (0.004±0.002 µM; Table 1), and it is notable that of all the agents tested, Dp44mT showed the greatest anti-proliferative efficacy.

**Table 1:** Anti-proliferative activity of the examined agents in MCF-7 cells.

MCF-7	IC_50_	Fe complex IC_50_	Cu complex IC_50_
	(µM)	(µM)	(µM)
DFO	16.902±5.726	391.855±201.062	
SIH	14.004±4.160	429.088±210.893**	
NHAPI	19.137±12.023	16.987±5.569	
Dp44mT	0.004±0.002	0.004±0.002	0.009±0.002
PTX	0.008±0.005		
5FU	47.912±12.992		
DOX	0.163±0.038		
MTX	3027.775±1105.490		
4HC	11.726±2.564		
TMX	10.773±1.923		

The studied agents (chelators and their fully coordinated iron (Fe) or copper (Cu) complexes and anti-cancer agents) were incubated with MCF-7 cells for 72 h at 37°C. Cell viability was determined using the neutral red uptake assay and the IC_50_ values (the concentration inducing a 50% reduction in proliferation compared to the control) were calculated using CalcuSyn 2.0 software; *n*≥4 experiments. Statistical significance (ANOVA): *p<0.05, **p<0.01, ***p<0.001 as compared to the same uncomplexed chelator.

The anti-proliferative activity of the Fe complexes of DFO and SIH were markedly (23–31-fold) decreased in comparison to those of the parent ligands alone (Table 1). These results correlate well with the anti-oxidative abilities of DFO and SIH that are known to form redox-inactive Fe complexes [18]. In contrast, NHAPI and Dp44mT displayed comparable anti-proliferative effects when assayed as free ligands or Fe complexes (Table 1 and Fig. S1). This was also the case for the Cu complex of Dp44mT, which showed similar anti-proliferative activity compared to the parent chelator (Fig. S1). These data are in good agreement with previous studies that demonstrate the ability of the Dp44mT-Fe or -Cu complexes to generate cytotoxic, reactive oxygen species [43]. Notably, the NHAPI-Fe complex has neither anti-oxidative nor pro-oxidative properties [18].

### 2. Combinations of Standard Anti-Cancer Agents with Chelators and their Iron Complexes Leads to a Wide Spectrum of Activity: From Strong Antagonism to Strong Synergism

#### 2.1. Combination of either DFO, SIH or NHAPI or their iron complexes with cytotoxic agents

The effects of combinations of anti-cancer drugs and Fe chelators or their metal-complexes on their IC_50_ values with regard to the proliferation of MCF-7 cells are shown in Fig. S3. The method of Chou and Talalay [23] was used to calculate values of the combination index (*CI*; Table 2).

**Table 2:** Quantitative assessments of the anti-proliferative activity of (A) combinations of metal-chelating agents and (B) their iron (Fe) and copper (Cu) complexes that were formed at an metal binding equivalent of 1 in MCF-7 cells.

Combination Index (*CI*)					
A	DFO	SIH	NHAPI	Dp44mT	
PTX	2.384±0.147	1.588±0.185	1.001±0.049	0.940±0.160	
5FU	1.708±0.361	1.140±0.417	0.607±0.105	0.309±0.115	
DOX	1.953±0.391	1.666±0.218	0.799±0.257	0.655±0.100	
MTX	1.706±0.072	1.542±0.438	0.981±0.157	1.315±0.320	
4HC	2.138±0.309	1.336±0.372	1.530±0.154	0.831±0.212	
TMX	1.540±0.679	0.396±0.153	0.688±0.203	0.494±0.172	
**B**	**DFO+Fe**	**SIH+Fe**	**NHAPI+Fe**	**Dp44mT+Fe**	**Dp44mT+Cu**
PTX	2.155±0.161	0.967±0.267	1.709±0.345	0.809±0.090	0.513±0.074
5FU	1.644±0.289	0.819±0.285	1.899±0.558***	0.304±0.088	0.162±0.013
DOX	1.104±0.039*	0.506±0.204***	2.057±0.598***	1.097±0.319	0.440±0.056
MTX	1.164±0.192	1.287±0.317	1.920±0.377**	0.681±0.285	0.386±0.185**
4HC	1.183±0.060**	1.452±0.509	1.378±0.357	0.707±0.052	0.690±0.015
TMX	1.146±0.122	1.456±0.211***	0.522±0.319	0.438±0.060	0.473±0.100

All of the studied compounds and their combinations were incubated with MCF-7 cells for 72 h at 37°C at a concentration corresponding to their IC_50_ value. The combination index (*CI*) values were calculated using the Chou and Talalay method from *n*≥4 experiments using CalcuSyn 2.0 software; *CI* <1, ≈1, or >1 indicate synergism, an additive effect, or antagonism, respectively. Statistical significance (ANOVA): *p<0.05, **p<0.01, ***p<0.001 as compared to the same combination with uncomplexed parent chelator.

Combinations of the six examined anti-neoplastic agents with DFO resulted in antagonistic effects with all studied anti-cancer drugs (from 1.540±0.679 with TMX to 2.384±0.147 with PTX; Table 2A). The effects of the Fe complex of DFO (Table 2B) in combination with most of the assayed anti-cancer drugs did not differ markedly from those of uncomplexed DFO. Nonetheless, complexation did result in a general decrease in the *CI* value for all combinations, leading to slight antagonism to antagonism. The only statistically significant (*p*<0.05) changes in *CI* values occured when the Fe complex of DFO was used in combination with DOX or 4HC (*CI*: 1.104±0.039 or 1.183±0.060, respectively; Table 2B), where slight antagonism occurred instead of the antagonism observed with uncomplexed DFO.

Combination of most of the anti-cancer drugs with SIH displayed slight antagonistic to antagonistic activity, with *CI* values ranging from 1.140±0.417 (5FU) to 1.666±0.218 (DOX), while the combination of SIH with TMX demonstrated synergism (*CI*: 0.396±0.153; Table 2A). Formation of the SIH-Fe complex significantly (*p*<0.001) reversed the interaction with DOX from antagonism to synergism (*CI*: 0.506±0.204), but also significantly (*p*<0.001) attenuated the synergism observed for the combination of SIH with TMX, leading to antagonism (*CI*: 1.456±0.211; Table 2B).

NHAPI exhibited nearly additive to synergistic anti-proliferative activity in combination with most of the anti-cancer drugs (Table 2A). In contrast, when NHAPI was combined with 4HC, antagonism (*CI*: 1.530±0.154) was identified. Use of the NHAPI-Fe complex resulted in an increases in the *CI* value when used in combination with PTX, 5FU, DOX, and MTX, leading to antagonism. In contrast, a slight, but not significant decrease in the *CI* occurred when the NHAPI-Fe complex was combined with 4HC and TMX, resulting in moderate antagonism and synergism, respectively (Table 2B).

#### 2.2. Combination of Dp44mT or its iron or copper complexes with cytotoxic agents

Due to the well documented ability of Dp44mT to form both Fe and Cu complexes that are important for their cytotoxic activity [35], [43], [44], both were examined in terms of combinations with cytotoxic agents relative to the ligand alone.

Combination of the anti-cancer drugs with the Dp44mT ligand alone yielded moderate antagonism with MTX, while for all other drugs, nearly additive or synergistic effects were observed, namely: nearly additive (PTX), moderate synergism (4HC) and synergism (TMX, DOX, and 5FU; Table 2A). The Dp44mT-Fe complex yielded results that were generally comparable to the uncomplexed parent chelator, and the *CI* value was either very similar, or lower for most of the combinations (Table 2B). The only exception was for the combination of Dp44mT-Fe complex with DOX, whereby a shift from synergism to nearly additive activity occurred.

Interestingly, the Dp44mT-Cu complex was the only agent that was shown to have consistently synergistic anti-proliferative properties with all six of the anti-cancer drugs that were assessed, with *CI* values ranging from 0.690±0.015 (synergism with 4HC) to 0.162±0.013 (strong synergism with 5FU; Table 2B).

#### 2.3. Chelator and cytotoxic drug interactions are dose-dependent

**Figure 2:**
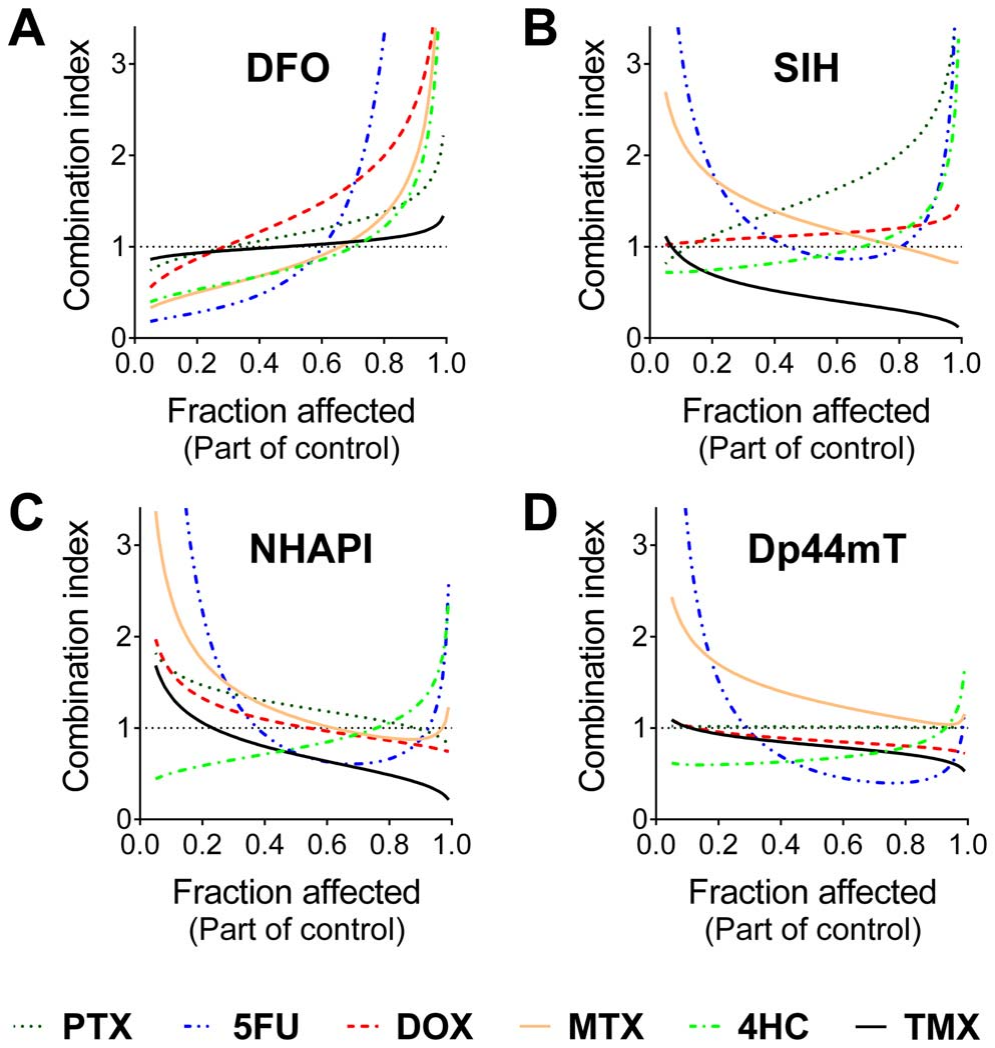
Chelator and anti-cancer drug interactions in MCF-7 cells as a function of activity. The computer simulations of the combination index (*CI*)-cellular proliferation (fraction affected - F*a*) dependency were obtained using CalcuSyn 2.0 software following a 72 h incubation of MCF-7 cells with combinations of the studied compounds at concentrations corresponding to their IC_50_ values and IC_50_ fractions and multiples (1/8, 1/4, 1/2, 1, 2, and 4). Results are means of *n*≥4 experiments.

As drug-drug interactions can change as their concentration changes [45], the combinations of chelators and anti-cancer drugs were also examined at fractions and multiples (1/8, 1/4, 1/2, 1, 2, and 4) of their IC_50_ values. The complete results are shown in Figs. S4, S5, and S6. From these data, the F*a*-*CI* plots were calculated using computer simulations (Fig. 2).

Our results indicated that the effects of Fe chelators on the anti-proliferative action of anti-cancer drugs were generally dose-dependent. Although DFO exhibited some synergism with the cytotoxic drugs at concentrations below the theoretical IC_50_ values of its combinations (*i.e.*, F*a* <0.5), there was a distinct shift toward antagonism with increasing dose (Fig. 2A).

Generally, for each of the other 3 chelators examined (*i.e.*, SIH, NHAPI and Dp44mT), the response as a function of F*a* was somewhat similar with each of the individual cytotoxic drugs except for PTX (Fig. 2B–D). Specifically, for the combinations of SIH with PTX, very low concentrations showed slight synergism with a rapid conversion to antagonism as the dose increased (Fig. 2B). For NHAPI and PTX, low and moderate concentrations were antagonistic with only very high concentrations leading to slight synergism (Fig. 2C). Interestingly, the combination of PTX and Dp44mT was nearly additive at all concentrations tested (Fig. 2D) and this behavior was unique amongst all of the combinations tested.

Although 5FU showed additive or even synergistic effects with most chelators when used at concentrations that were close to the respective IC_50_ values of the combinations, it displayed antagonistic effects at both extremely low and high concentrations (Fig. 2B–D). In the case of the combination of DOX and SIH, the effect was slightly different to the other 2 ligands, with a nearly additive response at low concentrations to an antagonistic effect at very high concentrations (Fig. 2B). Combination of DOX with NHAPI or Dp44mT led to similar behavior, with low doses being antagonistic or nearly additive, while high doses led to moderate synergism (Fig. 2C,D). For MTX, strong antagonism or antagonism was observed with all chelators at very low concentrations, while very high doses led to nearly additive activity (NHAPI and Dp44mT; Fig. 2C,D), or even moderate synergism (SIH; Fig. 2B). Combination of 4HC with either of NHAPI, SIH or Dp44mT led to synergism at low doses and this converted to an antagonistic effect at the highest doses (Fig. 2B–D). Finally, TMX was shown to have the most pronounced tendency to enhance synergism with increasing dose, irrespective of the particular chelator utilized (Fig. 2B–D). Hence, further studies were then initiated to examine the effect of combinations of NHAPI or Dp44mT with TMX. These agents were chosen for additional studies in other breast cancer cell-types as synergism was observed with the ligands and their metal complexes.

### 3. Synergism of Combinations of NHAPI or Dp44mT with TMX is Confirmed in the Estrogen Receptor Expressing T47D Cell Line, whereas an Antagonistic Effect of these Combinations was Observed using MDA-MB-231 Cells without the Estrogen Receptor

#### 3.1. Examination of the anti-proliferative activity of NHAPI, Dp44mT and their metal complexes or TMX in T47D and MD-MB-231 cells

To further assess the combinations of NHAPI or Dp44mT with the estrogen receptor antagonist, TMX [38], two more human breast carcinoma cell lines were used for comparison. The T47D human ductal breast carcinoma cell line expresses the estrogen receptor, as does the previously used MCF-7 human adenocarcinoma cell line [46]. In contrast, the MDA-MB-231 human adenocarcinoma cell line does not express the estrogen receptor [46]. Hence, these cell lines were used to understand the role of estrogen receptor expression on the potential synergistic activity between TMX and these chelators.

First, both the T47D (Fig. S7) and MDA-MB-231 (Fig. S8) cell lines were incubated with increasing concentrations of the studied agents for 72 h at 37°C to determine their IC_50_ values (Tables 3 and 4). In these studies, the T47D cells (Fig. S7A) were more susceptible to the Fe chelator, NHAPI, than MDA-MB-231 cells (Fig. S8A). However, the Fe complex of NHAPI had only slightly lower anti-proliferative activity than the ligand alone when assessing T47D cells (Table 3). The MDA-MB-231 cell line (Table 4) was more resistant to both Fe chelators and their metal complexes when compared to the MCF-7 (Table 1) and T47D cell lines (Table 3).

**Table 3:** Anti-proliferative activity of the examined agents in T47D cells.

T47D	IC_50_	Fe complex IC_50_	Cu complex IC_50_
	(µM)	(µM)	(µM)
NHAPI	7.037±0.786	10.434±4.590	
Dp44mT	0.002±0.000	0.003±0.001	0.003±0.001
TMX	5.659±0.686		

The studied agents (NHAPI and Dp44mT and their fully coordinated iron (Fe) or copper (Cu) complexes and tamoxifen (TMX)) were incubated with T47D cells for 72 h at 37°C. Cell viability was determined using the neutral red uptake assay and the IC_50_ values (the concentration inducing a 50% reduction in proliferation compared to the control) were calculated using CalcuSyn 2.0 software; *n*≥4 experiments. Statistical significance (ANOVA): *p<0.05, **p<0.01, ***p<0.001 as compared to the same uncomplexed chelator.

**Table 4:** Anti-proliferative activity of the examined agents in MDA-MB-231 cells.

MDA-MB-231	IC_50_	Fe complex IC_50_	Cu complex IC_50_
	(µM)	(µM)	(µM)
NHAPI	44.431±10.595	21.268±2.576***	
Dp44mT	0.055±0.028	0.047±0.022	0.046±0.017
TMX	5.821±0.529		

The studied agents (NHAPI and Dp44mT and their fully coordinated iron (Fe) or copper (Cu) complexes and tamoxifen (TMX)) were incubated with MDA-MB-231 cells for 72 h at 37°C. Cell viability was determined using the neutral red uptake assay and the IC_50_ values (the concentration inducing a 50% reduction in proliferation compared to the control) were calculated using CalcuSyn 2.0 software; *n*≥4 experiments. Statistical significance (ANOVA): *p<0.05, **p<0.01, ***p<0.001 as compared to the same uncomplexed chelator.

The metal complexes of NHAPI and Dp44mT demonstrated generally greater anti-proliferative activity against the MDA-MB-231 cell line than the chelators alone (Table 4), while this did not occur for T47D cells (Table 3). Surprisingly, and irrespective of their estrogen receptor status, both T47D and MDA-MB-231 cells showed similar IC_50_ values after incubation with TMX, *i.e.*, 5.7–5.8 µM (Tables 3, 4). On the other hand, MCF-7 cells, that are known for their autocrine stimulation of growth by estrogen [5], showed an IC_50_ value for TMX of 10.773±1.923 µM (Table 1).

#### 3.2. Combination of NHAPI or Dp44mT or their metal complexes with tamoxifen

The effects of combinations of anti-cancer drugs and chelators or their metal-complexes on their IC_50_ values with regard to the proliferation of MCF-7, T47D and MDA-MB-231 cells are shown in Figs. S9A and S10A The method of Chou and Talalay [23] was used to calculate values of the combination index (*CI*; Table 5).

**Table 5:** Quantitative assessments of the anti-proliferative activity of tamoxifen (TMX) combinations with (A) NHAPI and Dp44mT and (B) their iron (Fe) and copper (Cu) complexes that were formed at an metal binding equivalent of 1.

Combination Index (*CI*)			
A	NHAPI+TMX	Dp44mT+TMX	
MCF-7	0.688±0.203	0.494±0.172	
T47D	0.505±0.114	1.049±0.404	
MDA-MB-231	1.826±0.274	1.489±0.266	
**B**	**NHAPI+Fe**	**Dp44mT+Fe**	**Dp44mT+Cu**
	**+ TMX**	**+ TMX**	**+ TMX**
MCF-7	0.522±0.319	0.438±0.060	0.473±0.100
T47D	1.037±0.640	0.313±0.139	0.551±0.086
MDA-MB-231	1.574±0.387	1.765±0.382	1.754±0.263

All of the studied compounds and their combinations were incubated with MCF-7, T47D, or MDA-MB-231 cells for 72 h at 37°C at a concentration corresponding to their IC_50_ value. The combination index (*CI*) values were calculated using the Chou and Talalay method from *n*≥4 experiments using CalcuSyn 2.0 software; *CI* <1, ≈1, or >1 indicate synergism, an additive effect, or antagonism, respectively. Statistical significance (ANOVA): *p<0.05, **p<0.01, ***p<0.001 as compared to the same combination with uncomplexed parent chelator.

The combination of NHAPI with TMX resulted in synergism in MCF-7 and T47D cells, but antagonism in MDA-MB-231 cells (Table 5A). The combination of the NHAPI-Fe complex with TMX upon incubation of MCF-7 and MDA-MB-231 cells had a similar effect as the combination of NHAPI with TMX (Table 5B). However, using T47D cells, the synergistic effect of NHAPI in combination with TMX (Table 5A), shifted to an additive effect after incubation of these cells with the NHAPI-Fe complex and TMX (Table 5B).

The combination of Dp44mT with TMX was synergistic using MCF-7 cells, additive in T47D cells and antagonistic in the case of the MDA-MB-231 cell line (Table 5A). The complexes of Dp44mT with both Fe or Cu were synergistic in combination with TMX in MCF-7 and T47D cells (Table 5B). When assayed using MDA-MB-231 cells, the combinations of uncomplexed Dp44mT (Table 5A) as well as its metal complexes with TMX (Table 5B) had similar antagonistic effects.

#### 3.3. Chelator and cytotoxic drug interactions are dose-dependent

**Figure 3:**
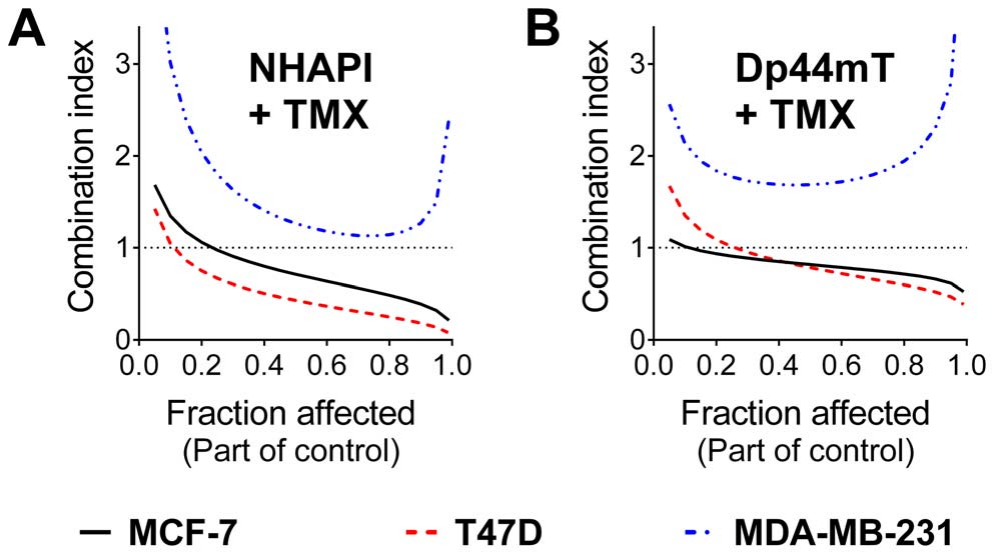
Interactions of NHAPI and Dp44mT with tamoxifen in MCF-7, T47D and MDA-MB-231 breast cancer cells as a function of activity. The computer simulations of the combination index (*CI*)-cellular proliferation (fraction affected - F*a*) dependency were obtained using CalcuSyn 2.0 software. These studies were performed using 72 h incubations of MCF-7, T47D, or MDA-MB-231 cells with combinations of the studied compounds at concentrations corresponding to their IC_50_ values and IC_50_ fractions and multiples (1/8, 1/4, 1/2, 1, 2, and 4). Results are means of *n*≥4 experiments.

The combinations of NHAPI or Dp44mT and TMX were then examined at fractions and multiples (1/8, 1/4, 1/2, 1, 2, and 4) of their IC_50_ values. The results using T47D and MDA-MB-231 cells are shown in Figs. S9B–C, and S10B–C. From these data, the F*a*-*CI* plots were calculated using computer simulations (Fig. 3).

Our results indicated that the estrogen receptor-expressing cell lines, MCF-7 and T47D, showed highly similar responses to the combinations of NHAPI or Dp44mT with TMX (Fig. 3A, B). In fact, at low doses (F*a*), antagonistic or moderate antagonistic values of *CI* were observed with the combination of NHAPI or Dp44mT and TMX using T47D cells, or NHAPI and TMX using MCF7 cells (Fig. 3A, B). In contrast, for the combination of Dp44mT with TMX using MCF-7 cells, nearly additive values of *CI* were observed at low doses (Fig. 3B). At higher doses, the *CI* values resulted in synergism (Dp44mT and TMX combination in MCF-7 and T47D; Fig. 3B) or even to strong synergism (NHAPI with TMX using MCF-7 cells; Fig. 3A) or very strong synergism (NHAPI with TMX on T47D cells; Fig. 3A).

In marked contrast to the results described above for the estrogen receptor-negative T47D and MCF-7 cell lines, the NHAPI or Dp44mT combinations with TMX on estrogen receptor-negative MDA-MB-231 cells resulted in wide U – shaped curves upon F*a*-*CI* simulation that displayed an antagonistic response (Fig. 3A, B).

### 4. Synergism of the Combination of NHAPI and Tamoxifen in MCF-7 cells is Confirmed by Measurement of Electrical Impedance, Mitochondrial Inner Membrane Potential and Cell Cycle Analyses

Since breast cancer combination therapy with some standard agents (*e.g.*, DOX) can lead to cardiotoxicity, it was important to determine which of the chelators would be most optimal to assess in more detailed investigations. Notably, NHAPI appears to show less potential for cardiotoxicity than SIH [31], while Dp44mT has demonstrated cardiotoxic effects at high, non-optimal doses [33]. Considering this, in addition to the marked synergism between TMX and NHAPI on two estrogen receptor-expressing cell lines (MCF-7 and T47D; Fig. 3A), further studies were initiated on the best observed combinations of TMX with NHAPI and the NHAPI-Fe complex using the MCF-7 cells. These investigations focused on cellular proliferation dynamics over time, cell cycle distribution, mitochondrial depolarization and cellular morphology. These experiments could potentially provide additional information in terms of the mechanism(s) of action.

**Figure 4:**
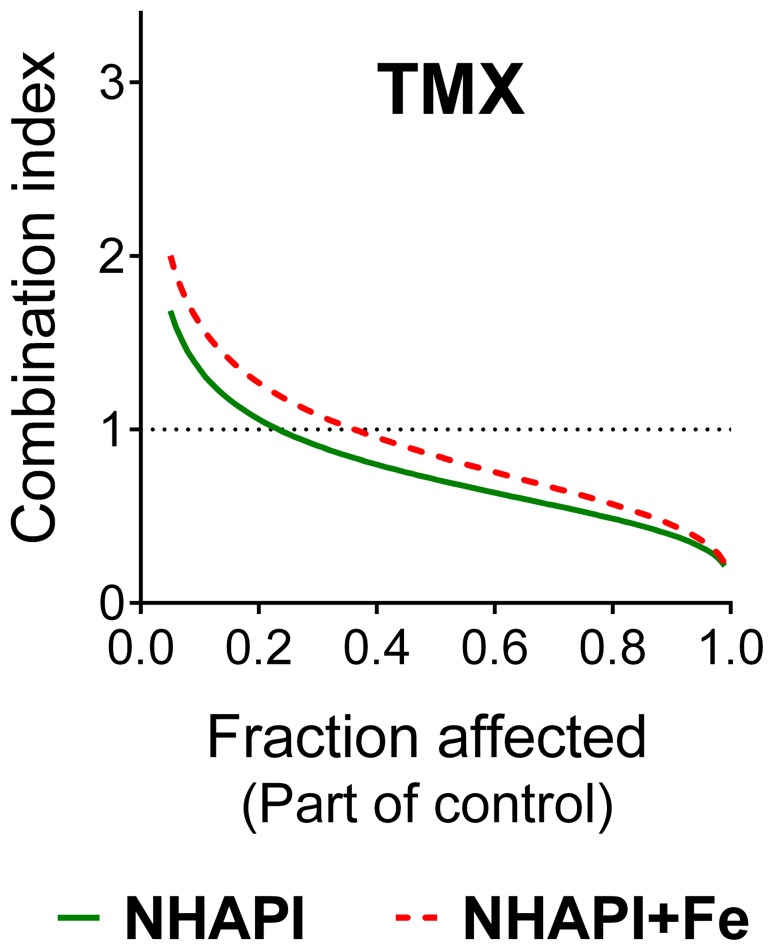
Combinations of NHAPI or its Fe complex with TMX in MCF-7 cells behave synergistically. The combination index-cellular proliferation dependency was calculated using CalcuSyn 2.0 software. These studies were performed using 72 h incubations of the studied agents at concentrations corresponding to the various IC_50_ multiples and fractions; *CI* <1, ≈1, or >1 indicate synergism, an additive effect, or antagonism, respectively. Results are means of *n*≥4 experiments.

First, the combination potential of the NHAPI-Fe complex with TMX was examined at various fractions and multiples (*i.e.*, 1/8, 1/4, 1/2, 1, 2 and 4) of their IC_50_ values. The generated *CI*–F*a* simulations of these combinations were very similar as with the parent uncomplexed chelator, NHAPI, and maintained the favorable trend of increasing synergism with increasing dose (Fig. 4). Hence, both the NHAPI ligand and iron complex were examined in further studies. In all of the results reported below, the following fractions and multiples of the IC_50_ of NHAPI or its iron complex and/or TMX were utilized, namely: 1/2, 1 and 2. For example, 2 NHAPI+Fe+TMX denotes the combination of NHAPI-Fe complex with TMX where both agents were added in concentrations corresponding to 200% of their IC_50_ values.

#### 4.1. Electrical impedance measurements

**Figure 5:**
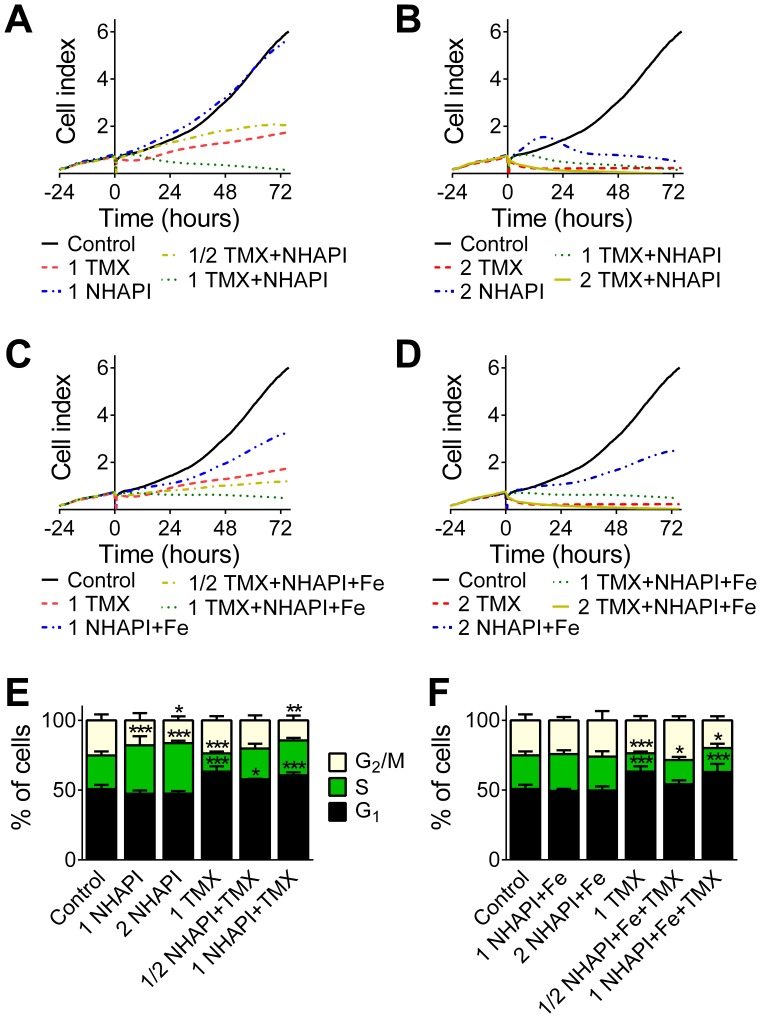
Combinations of NHAPI or its Fe complex with TMX caused decreases in proliferation and G_1_-S cell cycle arrest. The proliferation dynamics were measured as the electrical impedance of MCF-7 cells **(A, B, C and D)** and were monitored using an xCELLigence System for 72 h. Cell cycle analyses **(E and F)** were performed following a 72 h/37°C incubation with the agent using flow cytometry following propidium iodide staining. Results are: **(A–D)** means of *n*  = 3 experiments and **(E, F)** mean±SD (*n*≥4 experiments). Statistical significance (ANOVA): *p<0.05, **p<0.01, ***p<0.001 as compared to the control (untreated) group.

Combinations of NHAPI or the NHAPI-Fe complex with TMX were then assayed over 72 h using electrical impedance measurements of cellular proliferation *via* the xCELLigence System (see *Materials and Methods* for details). The cell adhesion rate assayed by this system is expressed as the “cell index” and parallels the viability or proliferation of the cells in real-time [42]. As observed in Fig. 5A,C, the inhibition of proliferation induced by the NHAPI-TMX combination was more pronounced than the effects of either of the individual agents alone. Moreover, different shapes of the proliferation curves of NHAPI, the NHAPI-Fe complex and their combinations with TMX were observed. The proliferation curve of NHAPI at its IC_50_ concentration (*i.e.*, 1 NHAPI) closely paralleled the control curve (Fig. 5A), which may be caused by an increase in cells in the S-phase of the cell cycle (see flow cytometric analysis described below in Fig. 5E) that have a broader adhesive area [47]. The shape of the proliferation curve of cells exposed to NHAPI at double its IC_50_ concentration (*i.e.*, 2NHAPI; Fig. 5B) consisted of an initial increase of adhesivity (above the control levels), followed later by decline in cellular viability (Fig. 5B).

#### 4.2. Flow cytometric analysis

Flow cytometric analyses revealed a slight, but significant (*p*<0.001) increase in the proportion of S-phase cells after a 72 h incubation with NHAPI (Fig. 5E). This observation is supported by the observed shape of the electrical impedance proliferation curve of the NHAPI-treated cells (Fig. 5B), as described above. Furthermore, a significant (*p*<0.05) decrease in G_2_/M-phase cells was observed following the incubation of the cells with the 2× IC_50_ concentration of NHAPI relative to the control (Fig. 5E). TMX at the tested concentration (1× IC_50_) showed a significantly (*p*<0.001) decreased S-phase population in favor of G_1_ cells. Increasing the concentration of TMX (to 2× IC_50_) resulted in excessive toxicity that precluded cell cycle analysis. In contrast to the ligand alone (Fig. 5E), the NHAPI-Fe complex caused no significant changes in the cell cycle (Fig. 5F). The combinations of NHAPI or its Fe complex with TMX resulted in a G_1_-S cell cycle arrest that was similar to that observed with TMX treatment alone (Fig. 5E and F).

#### 4.3. Mitochondrial membrane potential (ΔΨ_m_)

To examine the qualitative and quantitative assessment of the mitochondrial membrane potential (ΔΨ_m_), epifluorescence microscopy and flow cytometry were used implementing the JC-1 probe.

**Figure 6:**
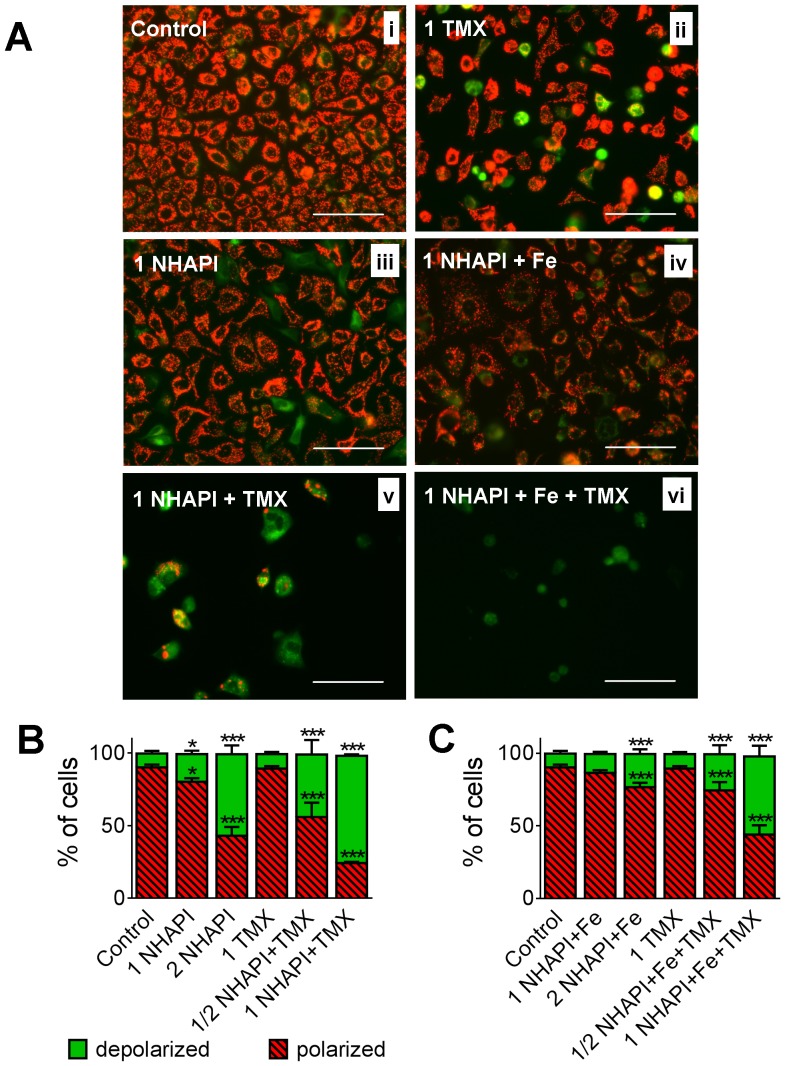
Dissipation of the inner mitochondrial membrane potential (ΔΨ_m_). **(A)** Fluorescence microscopy of MCF-7 cells stained with the JC-1 probe after 72 h incubations of MCF-7 cells with combinations of NHAPI or its Fe complex with TMX. The scale bars represent 100 µm. Phase-contrast photomicrographs showing morphology of the same cells are shown in Fig. S11. **(B, C)** Flow cytometric analyses of MCF-7 cells treated with combinations of NHAPI or its Fe complex with TMX were stained with the JC-1 probe. Results in **(A)** are typical of *n*≥4 experiments; **(B, C)** Mean±SD (*n*≥4 experiments). Statistical significance (ANOVA): *p<0.05, **p<0.01, ***p<0.001 as compared to the control (untreated) group.

Epifluorescence microscopy to assess ΔΨ_m_: Incubation of control cells with the JC-1 probe exhibited marked red fluorescence (Fig. 6Ai) which reflects the mitochondrial inner membrane potential (ΔΨ_m_)-dependent accumulation of probe dimers in actively respiring mitochondria [48]. Diffuse cytosolic green fluorescence indicates monomers of the probe released into the cytoplasm after mitochondrial depolarization, whereas yellow fluorescence indicates a mixture of red and green fluorescence in cells undergoing mitochondrial depolarization. A lack of fluorescence is indicative of probe release from necrotic or secondary apoptotic cells [48].

In cells incubated with TMX in particular, but also NHAPI and to a much lesser extent NHAPI-Fe, there was diffuse green and rarely yellow fluorescence (in TMX only) in some cells, but not others (Fig. 6Aii–iv). Combined incubations of NHAPI or the NHAPI-Fe complex with TMX resulted in a marked decrease in total fluorescence, which was primarily due to a loss of red fluorescence (Fig. 6Av, vi). In the small percentage of cells remaining after treatment with the combination of agents, green and sometimes yellow fluorescence was observed (Fig. 6Av, vi), with only an extremely minor indication of red fluorescence in the NHAPI+TMX combination (Fig. 6Av). Collectively, these observations from the combination studies are indicative of probe loss from necrotic or late-stage apoptotic cells due to organelle and membrane damage.

With respect to cellular morphology as shown by bright field microscopy, the cells that were incubated with TMX alone tended to retract their filopodia and “round up” (Fig. S11ii *cf.* i), whereas NHAPI slightly increased their surface area compared to the control cells (Fig. S11iii *cf.* i). Combinations of NHAPI or its Fe complex with TMX resulted in markedly more toxicity leading to fewer adherent cells (Fig. S11v & vi *cf.* i). In fact, at the light microscopy level, the cytotoxicity was more evident for NHAPI+Fe+TMX (Fig. S11vi) than NHAPI+TMX (Fig. S11v). As with the flow cytometry experiments, the 2 x IC_50_ concentrations of NHAPI+TMX, NHAPI+Fe+TMX and TMX groups resulted in virtual lack of cells available for documentation (see above), hence, these experiments could not be performed.

Flow Cytometric Analysis of Mitochondrial ΔΨ_m_: As an appropriate adjunct to the qualitative microscopic studies above, flow cytometric analysis was used to quantitatively assess ΔΨ_m_ of MCF-7 cells. After a 72 h incubation, the NHAPI ligand caused a significant (*p*<0.05 or *p*<0.001) increase in the number of cells with depolarized mitochondria at concentrations 1x and 2x its IC_50_, respectively (Fig. 6B). In contrast, only when the NHAPI-Fe complex was used at 2x its IC_50_ was there a significant (*p*<0.001) alteration in ΔΨ_m_ relative to the control (Fig. 6C). Indeed, at 1 x its IC_50_ concentration, the NHAPI-Fe complex (Fig. 6C), but also TMX (Fig. 6B), did not induce a significant change in ΔΨ_m_ compared to the control. In contrast, the combination of NHAPI or its Fe complex with TMX (at 1x their IC_50_ values) resulted in a significant (*p*<0.001) 50% decrease in the number of cells with polarized respiring mitochondria (Fig. 6B,C). Thus, these results from flow cytometric analysis confirmed the results of the epifluorescence microscopy and demonstrate the marked augmentation of the anti-proliferative activity of NHAPI or its Fe complex in combination with TMX.

## Discussion

One of the most important advances in pharmacological anti-cancer treatment is the use of combinations of agents with different mechanisms of action to achieve synergistic therapeutic effects with dose and toxicity reductions and to minimize or delay drug resistance [36], [37], [45]. Malignant cells typically require more Fe than normal cells to mediate rapid DNA synthesis and growth. Thus, depriving cancer cells of Fe is a promising new approach for anti-cancer chemotherapy [14], [49]. Iron deprivation causes a depletion of deoxyribonucleotides, which are produced from ribonucleotides by the Fe-dependent enzyme, ribonucleotide reductase (RR) [50].

Recent studies have identified other important molecules that modulate the cell cycle and cell signaling that are regulated by cellular Fe levels and are targets of Fe deprivation. These include members of the JNK and p38 MAPK signaling pathways [51], the CDK inhibitor, p21^CIP1/WAF1^ [20], the CDK inhibitor, p27^Kip1^ [52], check-point kinase, CHK1 [53], and the metastasis suppressor, NDRG1 [54], [55]. Of note, NDRG1 is regulated by the eukaryotic initiation factor 3a (eIF3a) during cellular stress caused by Fe depletion [56]. Furthermore, eIF3a also regulates the DNA repair ability of cancer cells and has been reported to influence their sensitivity to chemotherapeutics [57].

The vast majority of previous studies examining the anti-proliferative activity of Fe chelators have assessed their efficacy as single agents alone [14]–[17]. Hence, in the present investigation, we systematically assessed various drug combinations using a well-established median-effect analysis, also known as the “Chou-Talalay” method [23]. Notably, combined effects that are greater than each drug alone do not necessarily indicate synergism, as these results can be the result of an additive effect. Additionally, synergy and antagonism are very difficult to predict, which is particularly true for drugs with multiple mechanisms of action [23]. Hence, the current investigation was important due to the multiple molecular targets of Fe chelators [17], [58], their potential in cancer treatment [59] and the marked lack of information on the effect of combining these agents with standard chemotherapies.

The Fe chelators used clinically and in experimental biomedical research comprise a highly diverse group of agents that differ considerably in their physicochemical and biological properties [14], [58]. The bacterial siderophore, DFO, remains the “gold standard” for the treatment of Fe overload diseases [60], and the anti-cancer potential of DFO has been widely studied, due to its availability and favorable toxicological profile [24], [61]. However, DFO shows modest anti-tumor activity relative to other ligands [14], [62] and our results show that the interactions of DFO with clinically used anti-cancer drugs were the least favorable of all the assessed chelators. When examined at concentrations corresponding to its IC_50_ values, DFO was antagonistic when combined with all studied drugs (Table 2). Furthermore, the *CI* values of the DFO-containing combinations tended to increase with escalating dose, consistently shifting from synergism at low doses to antagonism at concentrations exceeding the IC_50_ value (Fig. 2A).

Pronounced hydrophilicity and high molecular weight are known to limit the plasma membrane permeability and efficacy of DFO [14], [63]. This ligand has been suggested to enter cells by an endocytotic mechanism [64], which may become saturated at higher concentrations and result in a plateau of its anti-proliferative efficacy. This was observed in combination experiments where the 1/2, 1, 2, and 4 multiples of the IC_50_ value of DFO induced comparable anti-proliferative activity (Figs. S4–S6). Formation of the DFO-Fe complex (ferrioxamine) markedly reduced the anti-proliferative properties of DFO alone, but it had no apparent effect on its combinatory potential. Hence, DFO displays modest anti-proliferative activity and no meaningful combinatory potential was observed with any of the chemotherapeutics.

In an effort to obtain more useful anti-cancer Fe chelators, a variety of novel agents have been obtained through chemical synthesis in recent years (reviewed in [14], [59], [65]). In this study, the more lipophilic tridentate aroylhydrazone chelators, SIH and NHAPI, showed anti-proliferative properties comparable to DFO when administered alone. However, their combinatory potential exhibited more promise as some synergistic combinations were identified. More importantly, the thiosemicarbazone ligand, Dp44mT, displayed significant anti-proliferative activity at concentrations that were three orders of magnitude lower than the three other chelators. Further, Dp44mT also displayed the best potential to act synergistically with established chemotherapeutics, further bolstering the marked potential of this class of ligands as anti-tumor agents [33], [44], [66], [67]. It is well known that Dp44mT forms redox-active Fe or Cu complexes that cause oxidative stress *via* the production of toxic ROS [14], [68]. Of note, synergism of the Dp44mT-Cu complex with the anti-neoplastic agents, gemcitabine or cisplatin, has recently been reported using the DMS-53 and A549 lung carcinoma cell lines [34]. However, Dp44mT can induce methemoglobin formation [67] and has also been reported to induce cardiac fibrosis following its administration at high, non-optimal doses [33].

Cardiotoxicity is a relatively common side effect of the chemotherapeutic agents that are used to treat breast cancer, such as DOX [21], [22]. Indeed, this drug induces irreversible, cumulative, dose-dependent cardiomyopathy and heart failure that may develop even years after successful breast cancer chemotherapy [21], [22] and can be further aggravated by the concomitant administration of PTX [69]. Therefore, the synergistic anti-proliferative combination of Dp44mT with DOX that was observed in the present study may also induce an increased risk of cardiotoxicity. This is of particular relevance considering that Dp44mT has previously shown no effect on inhibiting the cardiotoxic effects of DOX [70].

The breast cancer chemotherapeutics used in our combination studies with chelators differ considerably with respect to their mechanisms of action, which may account for the marked differences in their observed combinatory properties. In fact, PTX acts to stabilize microtubules during mitosis, although its activity may be antagonized by DNA synthesis inhibitors [38], such as the inhibition of RR caused by Fe depletion [71]. Hence, the nearly additive activity observed between PTX and Dp44mT may be the result of the redox-activity of its metal complexes, as Dp44mT was highly effective at nanomolar concentrations that are unlikely to induce pronounced Fe depletion.

The anti-metabolite, 5FU, in combination with SIH, NHAPI or Dp44mT at concentrations approximating their IC_50_ values, displayed an intriguing U-shaped dose response curve, with a rather narrow optimal concentration interval for synergism, but antagonism at higher or lower doses (Fig. 2B–D). Notably, 5FU is metabolized to 5-fluoro-2′-deoxyuridine-5′-phosphate (F-dUMP), an irreversible inhibitor of thymidylate synthase [38]. As this activation involves ribose reduction by RR [38], one of the targets of chelators [72], the synergistic effect may occur when the inhibition of RR is incomplete. Of interest, the sequential application of 5FU followed by Fe chelation has been previously suggested to be potentially beneficial because it normalized the increased serum Fe levels, and particularly the intra-myocardial Fe concentrations associated with 5FU-containing chemotherapy [73], [74].

Increased serum Fe levels have also been associated with high-dose MTX treatment [75], and considering this, Carmine *et al*. have recommended co-administration of Fe chelators with MTX therapy [75]. However, we found that combinations of chelators with MTX are antagonistic or, at best, nearly additive (Table 2). MTX inhibits dihydrofolate reductase, leading to depletion of the tetrahydrofolate co-factors that are required for purine and thymidine synthesis [38]. The simultaneous RR blockade mediated by chelators and the inhibition of another step in the deoxyribonucleotide biosynthetic pathway *via* MTX do not appear beneficial for the anti-proliferative activity observed on combination.

When assessing the DOX-chelator combinations, there were two antagonistic or moderately antagonistic interactions (with DFO and SIH) and two synergistic interactions (NHAPI and Dp44mT). Notably, DOX has at least three potential mechanisms of anti-cancer activity, namely: **(1)** it intercalates into DNA; **(2)** it is a topoisomerase II poison; and **(3)** it may form redox-active Fe complexes that promote toxic hydroxyl radical formation [38]. According to the current prevailing hypothesis, Fe-catalyzed ROS formation may be a possible cause of DOX cardiotoxicity [21], [22]. Both DFO and SIH are chelators with a documented ability to prevent oxidative stress and anthracycline cardiotoxicity [30], [76]. On the contrary, Dp44mT forms redox-active Fe and Cu complexes that promote ROS formation [43], [77]. Thus, the synergism between DOX and Dp44mT may be explained by the mutual potentiation of intracellular oxidative stress and DNA strand breaks. This hypothesis is in good agreement with the potentiation of synergism observed when combining DOX with the potently redox active Dp44mT-Cu complex [43], [77] (Table 2). However, it does not explain why the combination of the Dp44mT-Fe complex that displays redox activity [35], [44] and DOX led to nearly additive activity, while Dp44mT and DOX resulted in synergism.

The administration of Fe chelators has also been suggested following high-dose cyclophosphamide chemotherapy to manage increased serum non-transferrin-bound Fe that may be deleterious to health [78]. The active cyclophosphamide metabolite, 4HC, covalently attaches alkyl groups to the guanine N7 position and forms cross-links both between and within DNA strands [38]. Our results for the combinations of all chelators with 4HC ranged from synergism at lower F*a* values to antagonism at higher F*a* values.

Clearly, the most effective combinations in this study were observed with TMX. All of the chelators were synergistic using MCF-7 cells, except DFO, which showed antagonistic effects. Furthermore, TMX showed relatively constant dependence of *CI* on F*a* and displayed increasing synergism on two estrogen receptor-expressing cell lines (MCF-7 and T47D) with increasing dose (F*a*) of SIH (studied only with MCF-7 cells), NHAPI or Dp44mT (Figs. 2,3). The combination of NHAPI and Dp44mT with TMX using the estrogen receptor negative MDA-MB-231 cell line was constantly antagonistic throughout the entire F*a* scale, potentially due to the lack of the TMX target in these cells. Importantly, TMX is mostly indicated in breast cancer patients as a chemopreventive agent during the post-treatment period to prevent recurrence, as it competes with estradiol for estrogen receptor binding [79]. TMX also decreases the production of transforming growth factor *β* (TGF- *β*) and insulin-like growth factor 1 (IGF-1) that can act to stimulate oncogenesis [38]. Interestingly, MCF-7 cells have been reported to synthesize their own transferrin in an estrogen-dependent manner that may act in an autocrine fashion to stimulate Fe uptake by tumor cells, and thus, proliferation [5], [7]. Hence, the synergism between TMX and Fe chelators may be caused by potentiation of Fe depletion-induced by TMX blocking estrogen-induced Tf synthesis and also direct Fe-binding by the ligands. Synergism between TMX and inositol hexaphosphate (IP6), a naturally-occurring polyphosphorylated carbohydrate with Fe-chelating properties, has also been previously reported in MCF-7 cells [80].

Of the combinations of chelators with chemotherapeutics examined in the current study, the most promising combination particularly in terms of maximizing efficacy and minimizing the problem of cardiotoxicity, was considered to be NHAPI or its Fe complex with TMX. This combination was utilized due to the marked synergism observed (Fig. 4) and that NHAPI in contrast to Dp44mT does not appear to result in cardiotoxicity [31]. The combinations of NHAPI or the NHAPI-Fe complex with TMX resulted in G_1_-S cell cycle arrest, and their synergism was further confirmed by the cellular morphology analyses and ΔΨ_m_ measurements. In regard to these latter studies on mitochondrial membrane potential, it is notable that mitochondria are of central importance in apoptotic cell death as well as the major sites of Fe metabolism, being critical for heme and iron-sulfur cluster synthesis (reviewed in [81]). Hence, the marked effect of the NHAPI and TMX combination on this latter organelle was important to assess and demonstrated that it was potently affected. Further detailed studies on the effect of these agents on mitochondrial metabolism are critical to further investigate, as it appears to be a key target.

In conclusion, this is the first systematic study to quantitatively assess interactions between Fe chelators and established chemotherapeutic agents using breast cancer cells. The combinations led to antagonism, but also synergism, with the effects observed being dose-dependent. Collectively, our results indicate that synergistic interactions are observed with combinations of the lipophilic chelators, NHAPI or Dp44mT, with DOX and also the combinations of SIH, NHAPI or Dp44mT with TMX. Furthermore, our data encourage further research of the combination potential of TMX with Fe chelation and strengthen the hypothesis regarding the Fe – estrogen link in breast cancer. The results are important for on-going preclinical studies with these novel Fe-chelating agents and also for future clinical trials.

## Supporting Information

Figure S1 **Effect of increasing concentration of the following metal salts or chelators on the proliferation of MCF-7 cells following 72 h incubations: (A) Ferric ammonium citrate; (B) Cu sulfate; (C) DFO; (D) SIH; (E) NHAPI; (F) and Dp44mT.** For **(C, D, E, and F),** the effect of the chelators are compared to their Fe and/or Cu complexes. Notably, the 1 and 3 µM concentrations of the DFO+Fe group induced no anti-proliferative activity. Results are mean±SD (*n*≥4 experiments). Statistical significance (ANOVA): *p<0.05, **p<0.01, ***p<0.001 as compared to the control (untreated) group.
(TIF)

Figure S2 **Effect of increasing concentration of anti-neoplastic agents on the proliferation of MCF-7 cells following 72 h incubations. (A)** PTX; **(B)** 5FU; **(C)** MTX; **(D)** DOX; **(E)** 4HC; and **(F)** TMX. Results are mean±SD (*n*≥4 experiments). Statistical significance (ANOVA): *p<0.05, **p<0.01, ***p<0.001 as compared to the control (untreated) group.
(TIF)

Figure S3 **Effect of the chelators, their Fe or Cu, complexes, the anti-neoplastic agents, or their combinations on the proliferation of MCF7 cells after a 72 h incubation at 37**°**C.** All of the studied agents and their combinations were incubated with MCF-7 cells at concentrations corresponding to their IC_50_ values. Results are mean±SD (*n*≥4 experiments). Statistical significance (ANOVA): *p<0.05, **p<0.01, ***p<0.001 as compared to the control (untreated) group.
(TIF)

Figure S4 **The anti-proliferative effects of either: the iron chelators alone (DFO, SIH, NHAPI, Dp44mT), the anti-neoplastic agents alone (PTX or 5FU), or their combinations at concentrations corresponding to their IC_50_ values and IC_50_ fractions and multiples (1/8, 1/4, 1/2, 1, 2, and 4) on MCF7 cells after a 72 h incubation at 37**°**C. (A, B)** DFO; **(C, D)** SIH; **(E, F)** NHAPI; **(G, H)** Dp44mT**; (A, C, E, G)** PTX; or **(B, D, F, H),** 5FU and their combinations. Results are mean±SD **(** *n*≥4 experiments). Statistical significance (ANOVA): *p<0.05, **p<0.01, ***p<0.001 as compared to the control (untreated) group.
(TIF)

Figure S5 **The anti-proliferative effects of either: the iron chelators alone (DFO, SIH, NHAPI, Dp44mT), the anti-neoplastic agents alone (MTX or DOX), or their combinations at concentrations corresponding to their IC_50_ values and IC_50_ fractions and multiples (1/8, 1/4, 1/2, 1, 2, and 4) on MCF7 cells after a 72 h incubation at 37°C.** **(A, B)** DFO; **(C, D)** SIH; **(E, F)** NHAPI; **(G, H)** Dp44mT**; (A, C, E, G)** MTX or **(B, D, F, H**), DOX and their combinations. Results are mean±SD (*n*≥4 experiments). Statistical significance (ANOVA): *p<0.05, **p<0.01, ***p<0.001 as compared to the control (untreated) group.
(TIF)

Figure S6 **The anti-proliferative effects of either: the iron chelators alone (DFO, SIH, NHAPI, Dp44mT), the anti-neoplastic agents alone (4HC or TMX), or their combinations at concentrations corresponding to their IC_50_ values and IC_50_ fractions and multiples (1/8, 1/4, 1/2, 1, 2, and 4) on MCF7 cells after a 72 h incubation at 37**°**C. (A, B)** DFO; **(C, D)** SIH; **(E, F)** NHAPI; **(G, H)** Dp44mT**; (A, C, E, G)** 4HC or **(B, D, F, H),** TMX and their combinations. Results are mean±SD (*n*≥4 experiments). Statistical significance (ANOVA): *p<0.05, **p<0.01, ***p<0.001 as compared to the control (untreated) group.
(TIF)

Figure S7 **Effect of increasing concentrations of: (A) NHAPI or its iron complex (Ligand: Metal –2∶1); (B) Dp44mT or its iron(III) and copper(II) complexes (Ligand: Metal –2∶1); and (C) Tamoxifen (TMX); on the proliferation of T47D cells following 72 h incubations at 37°C.** Results are mean ± SD (n≥4 experiments). Statistical significance (ANOVA): *p<0.05; **p<0.01; ***p<0.001 as compared to the control (untreated) group.
(TIF)

Figure S8 **Effect of increasing concentrations of: (A) NHAPI or its iron complex (Ligand: Metal –2∶1); (B) Dp44mT or its iron(III) and copper(II) complexes (Ligand: Metal –2∶1); and (C) Tamoxifen (TMX); on the proliferation of MDA-MB 231 cells following 72 h incubations at 37°C.** Results are mean ± SD (n≥4 experiments). Statistical significance (ANOVA): *p<0.05; **p<0.01; ***p<0.001 as compared to the control (untreated) group.
(TIF)

Figure S9 **The anti-proliferative effects of: (A) NHAPI or its iron complex (Ligand: Metal –2∶1), and Dp44mT or its iron(III) and copper(II) complexes (Ligand: Metal –2∶1) in combination with Tamoxifen (TMX) in their IC_50_ concentrations; (B) NHAPI, Tamoxifen (TMX) or their combinations; and (C) Dp44mT, Tamoxifen (TMX) or their combinations at concentrations corresponding to their IC_50_ values and IC_50_ fractions and multiples (1/8, 1/4, 1/2, 1, 2, and 4); on the proliferation of T47D cells following 72 h incubations at 37°C.** Results are mean+SD (n≥4 experiments). Statistical significance (ANOVA): *p<0.05, **p<0.01, ***p<0.001 as compared to the control (untreated) group.
(TIF)

Figure S10 **The anti-proliferative effects of: (A) NHAPI or its iron complex (Ligand: Metal –2∶1), and Dp44mT or its iron(III) or copper(II) complexes (Ligand: Metal –2∶1) in combination with Tamoxifen (TMX) in their IC_50_ concentrations; (B) NHAPI, Tamoxifen (TMX) or their combinations; and (C) Dp44mT, Tamoxifen (TMX) or their combinations at concentrations corresponding to their IC_50_ values and IC_50_ fractions and multiples (1/8, 1/4, 1/2, 1, 2, and 4); on the proliferation of MDA-MB-231 cells following 72 h incubations at 37°C.** Results are mean±SD (n≥4 experiments). Statistical significance (ANOVA): *p<0.05, **p<0.01, ***p<0.001 as compared to the control (untreated) group.
(TIF)

Figure S11 **Cellular morphology of MCF-7 cells following treatment with TMX, NHAPI, the NHAPI-Fe complex, or their combinations at concentrations corresponding to their IC_50_ values after a 72 h incubation at 37°C.** The scale bars represent 100 µm. Epifluorescence microscopy of ΔΨ_m_ analysis for the same cells are shown in Fig. 5A. Concentrations of agents are expressed as multiples of their IC_50_ values (*i.e.* 1 NHAPI+Fe+TMX denotes combination of the NHAPI-Fe complex with TMX, where both compounds were added at the concentration corresponding to their IC_50_ values).
(TIF)
