# Supplementary figures and images for: Quantitative Analysis of the Anti-Proliferative Activity of Combinations of Selected Iron-Chelating Agents and Clinically Used Anti-Neoplastic Drugs
Source: PLoS One. 2014 Feb 20;9(2):e88754. doi: 10.1371/journal.pone.0088754 (PMC3930662; doi:10.1371/journal.pone.0088754)

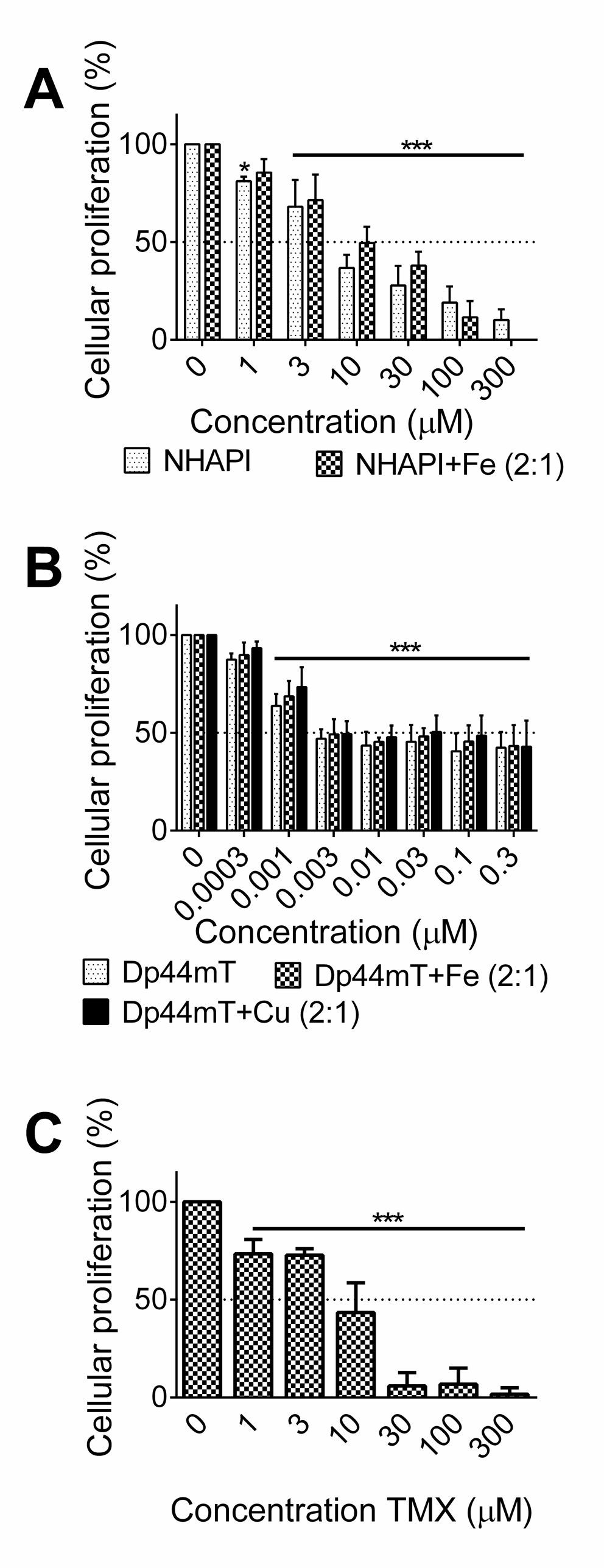

Supplement: Figure S7 — Effect of increasing concentrations of: (A) NHAPI or its iron complex (Ligand: Metal –2∶1); (B) Dp44mT or its iron(III) and copper(II) complexes (Ligand: Metal –2∶1); and (C) Tamoxifen (TMX); on the proliferation of T47D cells following 72 h incubations at 37°C. Results are mean ± SD (n≥4 experiments). Statistical significance (ANOVA): *p<0.05; **p<0.01; ***p<0.001 as compared to the control (untreated) group. (TIF) [file pone.0088754.s007.tif]

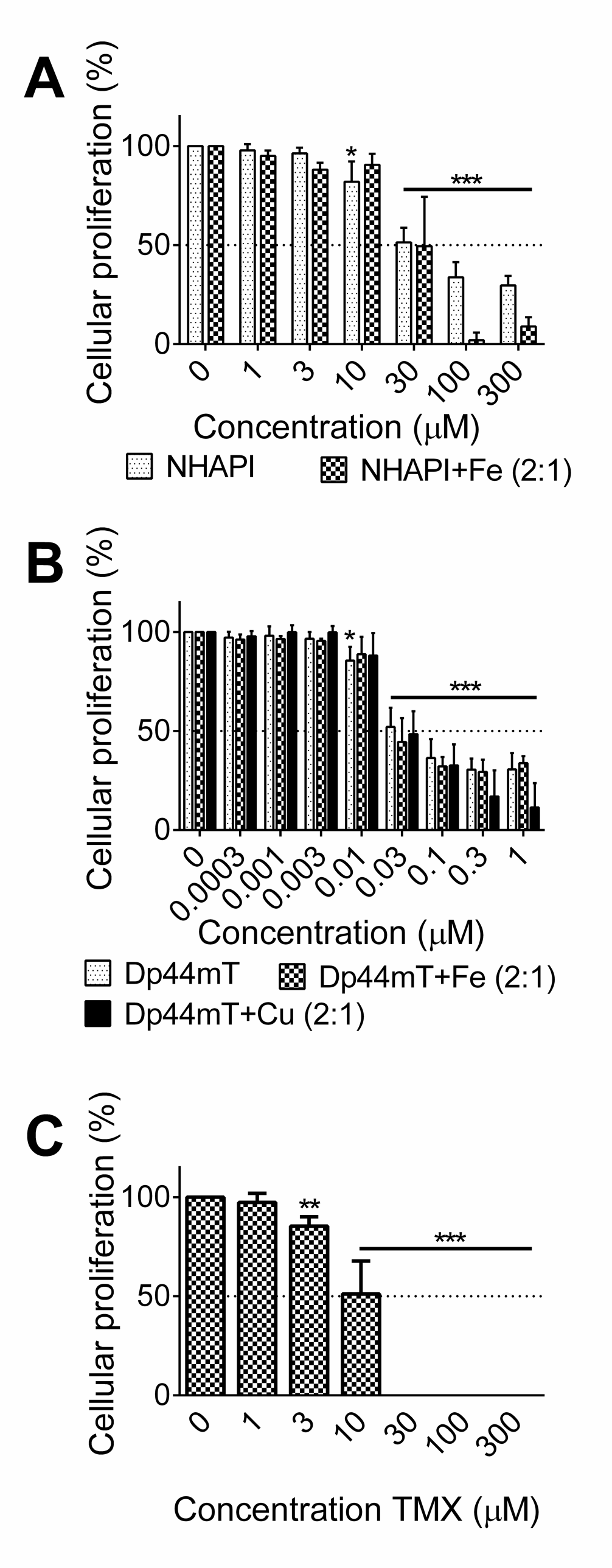

Supplement: Figure S8 — Effect of increasing concentrations of: (A) NHAPI or its iron complex (Ligand: Metal –2∶1); (B) Dp44mT or its iron(III) and copper(II) complexes (Ligand: Metal –2∶1); and (C) Tamoxifen (TMX); on the proliferation of MDA-MB 231 cells following 72 h incubations at 37°C. Results are mean ± SD (n≥4 experiments). Statistical significance (ANOVA): *p<0.05; **p<0.01; ***p<0.001 as compared to the control (untreated) group. (TIF) [file pone.0088754.s008.tif]

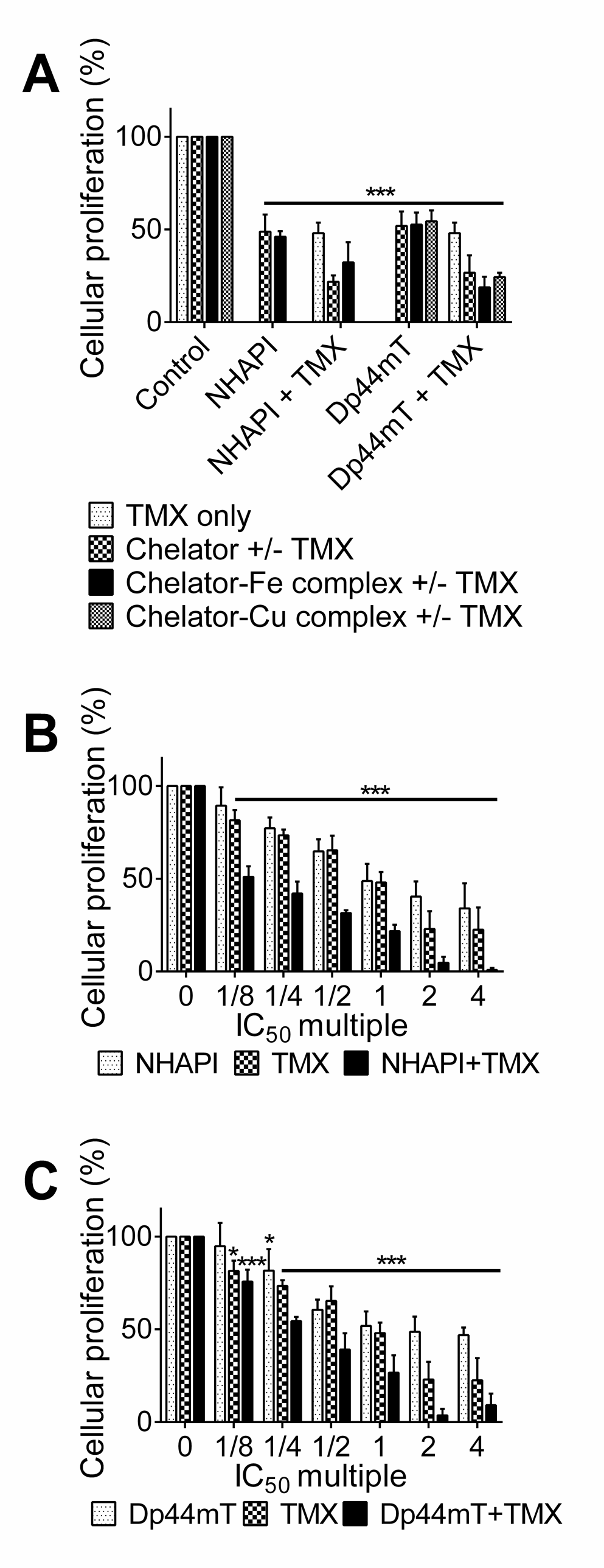

Supplement: Figure S9 — The anti-proliferative effects of: (A) NHAPI or its iron complex (Ligand: Metal –2∶1), and Dp44mT or its iron(III) and copper(II) complexes (Ligand: Metal –2∶1) in combination with Tamoxifen (TMX) in their IC50 concentrations; (B) NHAPI, Tamoxifen (TMX) or their combinations; and (C) Dp44mT, Tamoxifen (TMX) or their combinations at concentrations corresponding to their IC50 values and IC50 fractions and multiples (1/8, 1/4, 1/2, 1, 2, and 4); on the proliferation of T47D cells following 72 h incubations at 37°C. Results are mean+SD (n≥4 experiments). Statistical significance (ANOVA): *p<0.05, **p<0.01, ***p<0.001 as compared to the control (untreated) group. (TIF) [file pone.0088754.s009.tif]

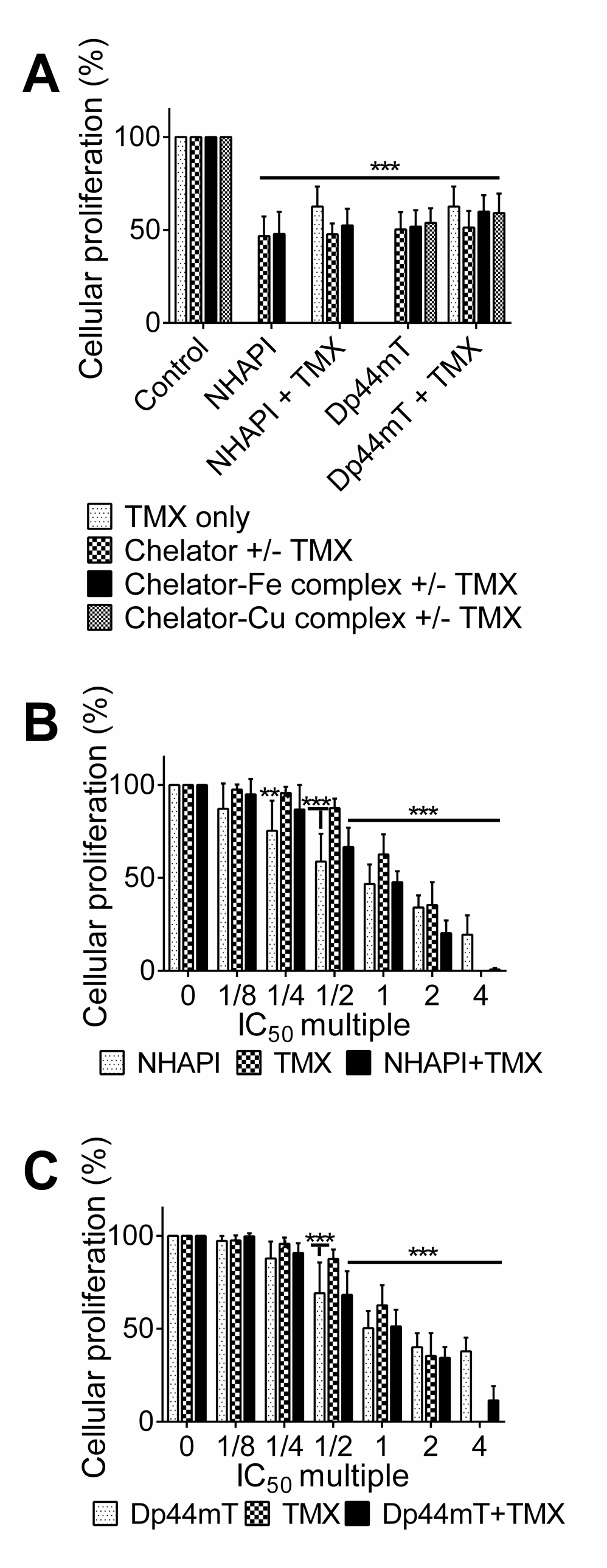

Supplement: Figure S10 — The anti-proliferative effects of: (A) NHAPI or its iron complex (Ligand: Metal –2∶1), and Dp44mT or its iron(III) or copper(II) complexes (Ligand: Metal –2∶1) in combination with Tamoxifen (TMX) in their IC50 concentrations; (B) NHAPI, Tamoxifen (TMX) or their combinations; and (C) Dp44mT, Tamoxifen (TMX) or their combinations at concentrations corresponding to their IC50 values and IC50 fractions and multiples (1/8, 1/4, 1/2, 1, 2, and 4); on the proliferation of MDA-MB-231 cells following 72 h incubations at 37°C. Results are mean±SD (n≥4 experiments). Statistical significance (ANOVA): *p<0.05, **p<0.01, ***p<0.001 as compared to the control (untreated) group. (TIF) [file pone.0088754.s010.tif]

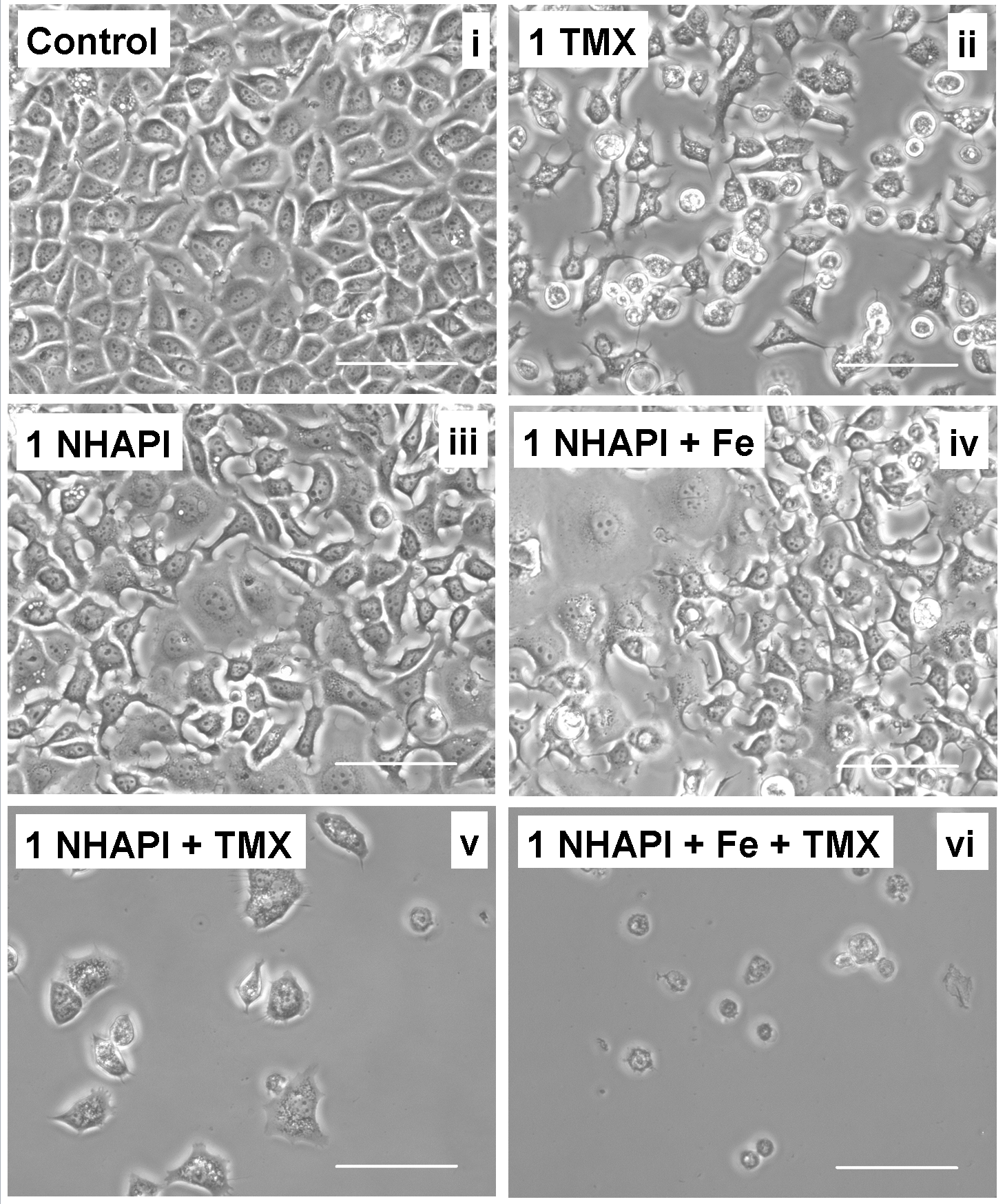

Supplement: Figure S11 — Cellular morphology of MCF-7 cells following treatment with TMX, NHAPI, the NHAPI-Fe complex, or their combinations at concentrations corresponding to their IC50 values after a 72 h incubation at 37°C. The scale bars represent 100 µm. Epifluorescence microscopy of ΔΨm analysis for the same cells are shown in Fig. 5A. Concentrations of agents are expressed as multiples of their IC50 values (i.e. 1 NHAPI+Fe+TMX denotes combination of the NHAPI-Fe complex with TMX, where both compounds were added at the concentration corresponding to their IC50 values). (TIF) [file pone.0088754.s011.tif]
